# 4292 Epigenetic Age and Depressive Symptoms in African American Women with Cardiometabolic Conditions

**DOI:** 10.1017/cts.2020.331

**Published:** 2020-07-29

**Authors:** Nicole B Perez, Allison Vorderstrasse, Gary Yu, Jacquelyn Taylor

**Affiliations:** 1 New York University General Clinical Research Center

## Abstract

OBJECTIVES/GOALS: To examine the relationship between epigenetic age acceleration (EAA) and depressive symptoms in a cohort of African American women (AAW) with cardiometabolic conditions (CMC) including hypertension, diabetes, obesity; and to explore clinical phenotypes of depressive symptoms in this population. METHODS/STUDY POPULATION: This secondary analysis utilized genomic and longitudinal clinical data from AAW in the InterGEN cohort (n = 250). EWAS data was used to estimate EAA based on the Horvath method, which incorporates the DNA methylation statuses at 353 specific CpG sites and regresses this epigenetic age on chronological age to determine EAA. Pearson’s correlations and linear regression will be used to examine the relationship between EAA and depressive symptoms and a linear mixed model will investigate this relationship over four time points during a two-year period. Clinical phenotyping of depressive symptoms will be explored using a cluster analysis. RESULTS/ANTICIPATED RESULTS: Analysis is underway and will be complete by the time of presentation. We hypothesize that higher EAA will associate with higher depressive symptoms and poorer trajectories over time. We expect that this relationship may be meditated by the presence of CMCs. Exploratory analysis of clinical phenotyping is expected to provide descriptive evidence with respect to specific depressive symptoms or clusters which are most associated with EAA and CMCs. These results will address several gaps. To our knowledge, this is the first study to examine the relationship of EAA and depressive symptoms considering the role of CMC, in a historically understudied population with disproportionate risk. DISCUSSION/SIGNIFICANCE OF IMPACT: Depression limits life quality and quantity and is highly comorbid in CMC. AAW have high risk of comorbidity, and this study furthers knowledge of depression and aging with a clinically accessible marker and aids recognition of a heterogenous phenotype in an undertreated population.

